# Supplementary figures and images for: Contemporary HIV-1 envelope pseudovirus panels for detecting and assessing B cell lineages with broadly neutralizing antibody potential
Source: PLoS Pathog. 2026 Apr 23;22(4):e1013739. doi: 10.1371/journal.ppat.1013739 (PMC13105343; doi:10.1371/journal.ppat.1013739)

# 119 pseudovirus global panel

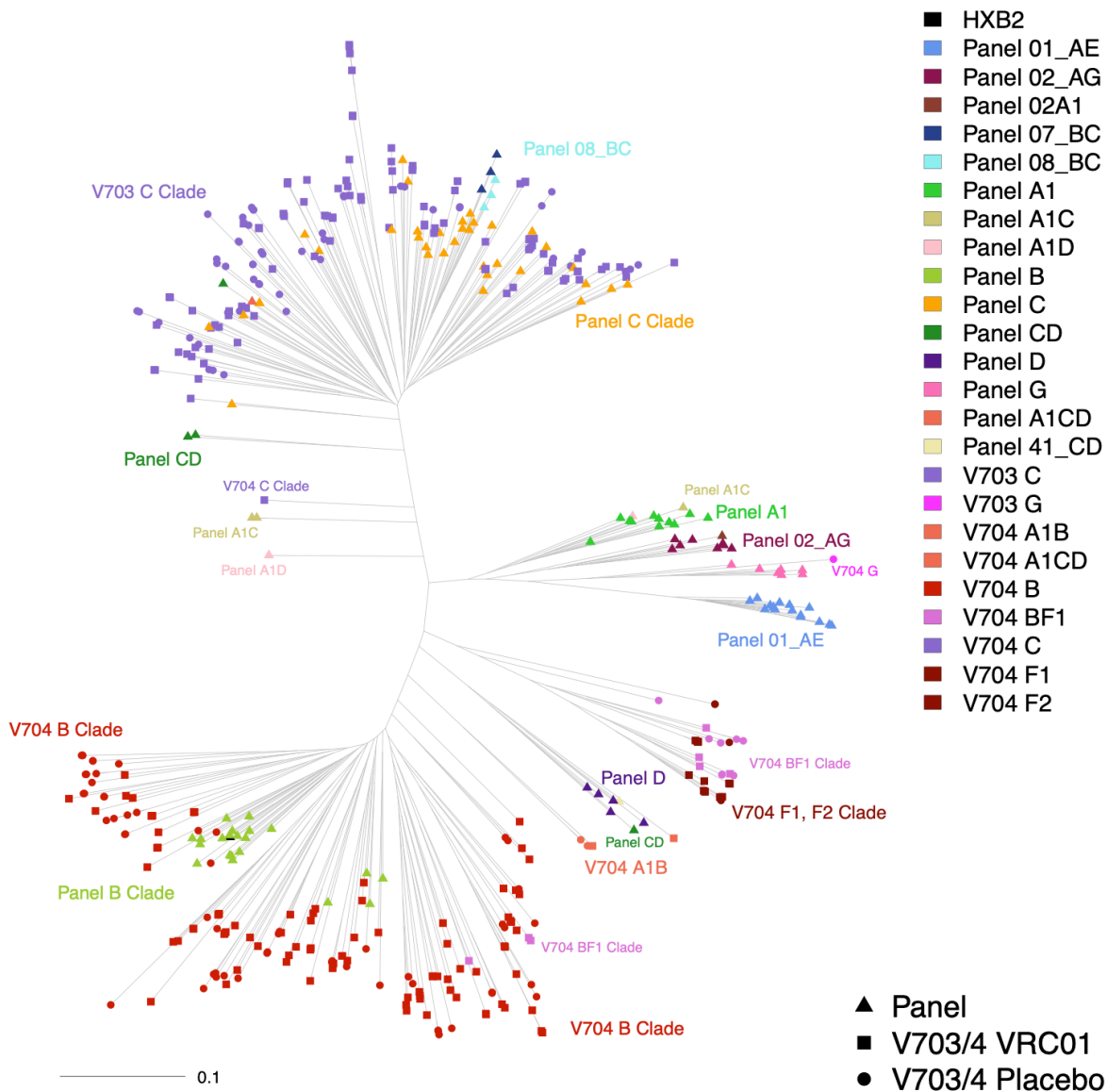

Supplement: S1 Fig — Phylogenetic analysis shows that within B and C clades the 119 viral panel sequences are not representative of the diversity sampled in the more contemporary AMP trial. Note that the exact composition of the panel can vary by a few viruses between studies. The phylogenetic tree was based on data from a version of the 119 virus panel that was downloaded using the CATNAP tool from the Los Alamos Database accessed on July 1, 2025 [7]. (PDF) [file ppat.1013739.s001.pdf]

# 200 pseudovirus C clade panel

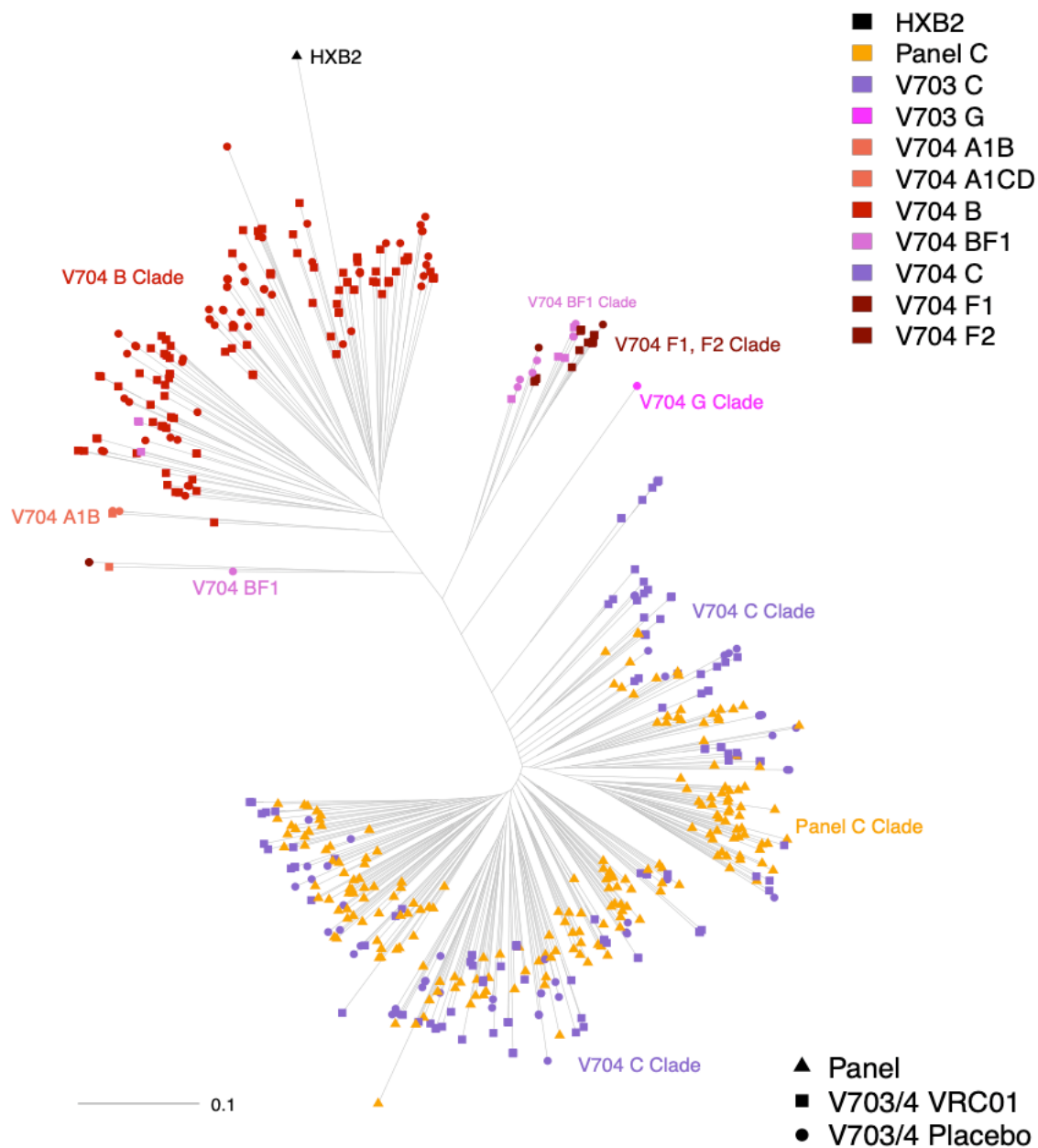

Supplement: S2 Fig — Of all commonly used panels described in this paper, this exclusively clade C panel, while lacking representation for all other clades, was the closest to contemporary C clade viruses sampled from the AMP V703 participants. Notably though, within the clade C subtree, AMP sequences tend to have longer branch lengths than the clade C panel pseudoviruses and are more spread out in the tree. The phylogenetic tree was based on data from a version of the 200 virus panel that was downloaded using the CATNAP tool from the Los Alamos Database accessed on July 1, 2025 [7]. (PDF) [file ppat.1013739.s002.pdf]

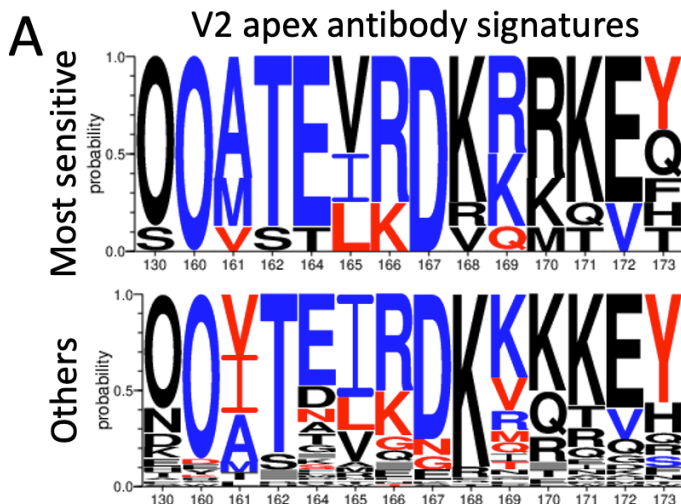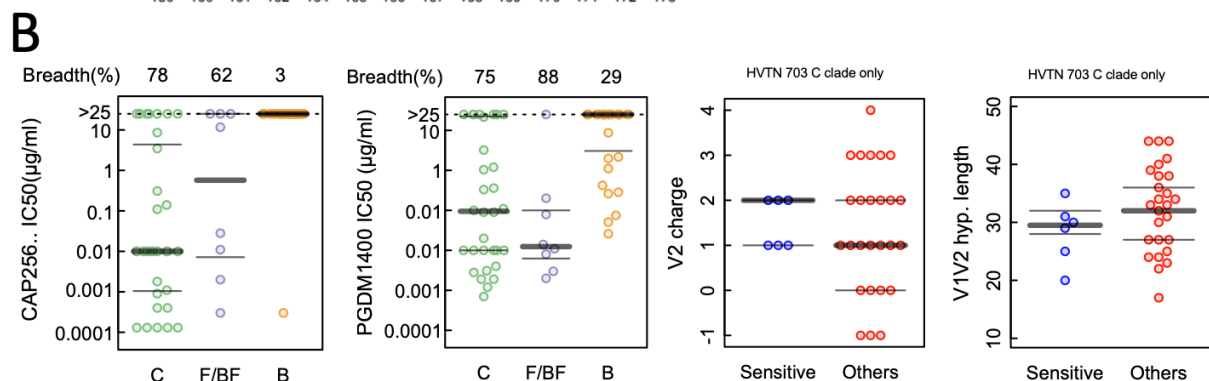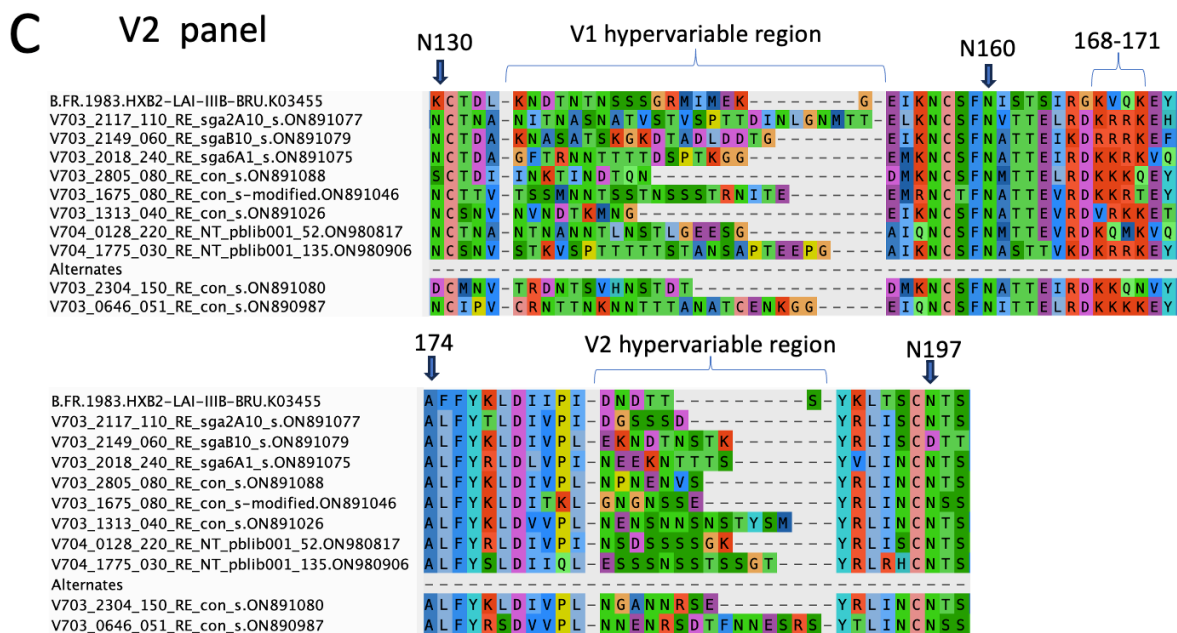

Supplement: S3 Fig — (A) The frequency of V2 apex bnAb sensitivity/resistance signatures as defined in Bricault et al. [34] in the class specific panel versus other AMP viruses. The height of the letter is indicative of the frequency of the amino acid in a given position in each group. An O indicates an N-linked glycosylation site. Blue are sensitivity signatures, red are resistance signatures, and black amino acids were not significantly associated with either one. (B) On the left we illustrate (also see S5 Table) the enhanced sensitivity of C clade and F/BF viruses relative to B clade viruses among the AMP collection of viruses to neutralization by the V2 apex bnAb CAP256 VRC26.25 LS (far left) and PGDM1400 (second to the left). On the right we show the distribution of hypervariable region characteristics that were associated with V2 apex bnAb neutralization [34]. As B clade viruses tend to be resistant regardless of loop characteristics, we restricted these comparisons to AMP C clade viruses. Positive V2 charge and shorter combined V1V2 hypervariable region lengths are signatures of sensitivity to V2 Apex antibodies [34], and V2 hypervariable loop positive charge and shorter hypervariable regions are slightly enriched in the sensitive panel. (C) The sequence alignment for the V2 apex bnAb sensitive panel across the epitope region. (PDF) [file ppat.1013739.s003.pdf]

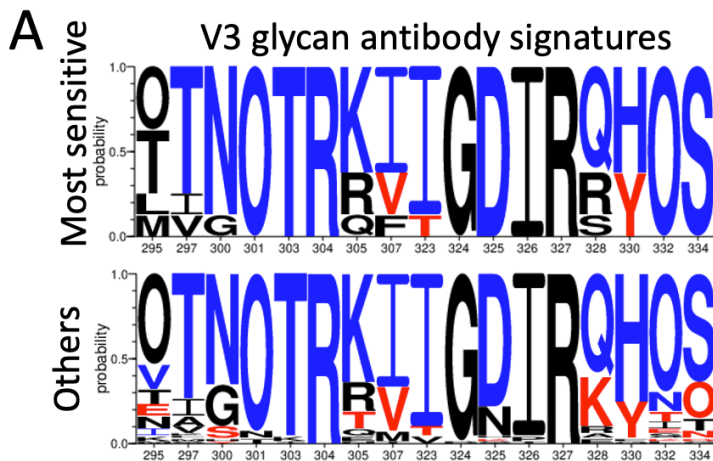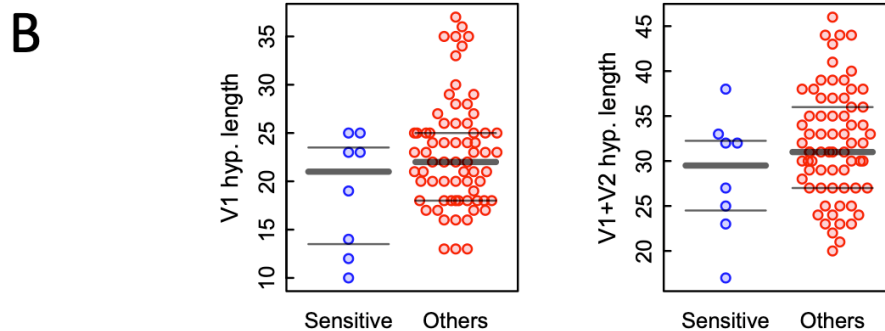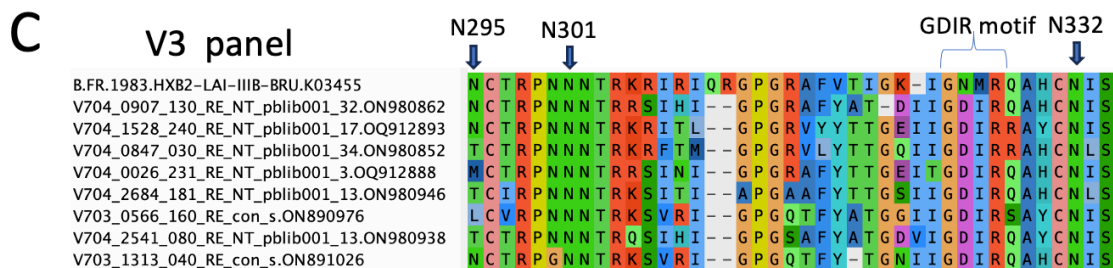

Supplement: S4 Fig — (A) The frequency of V3 glycan bnAb sensitivity/resistance signatures as defined in Bricault et al. [34] in the class specific panel versus other AMP viruses. The height of the letter is indicative of the frequency of the amino acid in a given position in each group. An O indicates an N-linked glycosylation site. Blue are sensitivity signatures, red are resistance signatures, and black amino acids were not significantly associated with either one. (B) Shorter V1 hypervariable length and shorter combined V1V2 hypervariable region lengths are associated with enhanced sensitivity to V3 glycan bnAbs [34], and there is an enrichment for shorter V1 and V1 + V2 hypervariable regions in the V3 glycan sensitivity panel. (C) The sequence alignment for the V3 glycan bnAb sensitive panel across the epitope region. (PDF) [file ppat.1013739.s004.pdf]

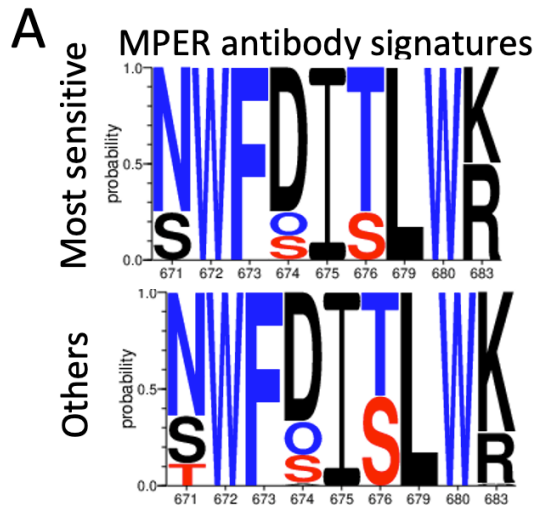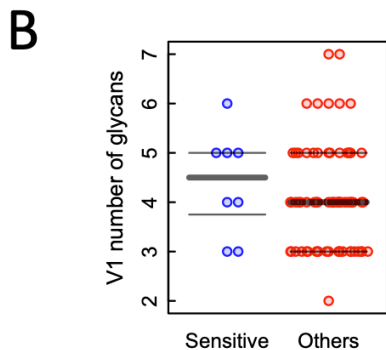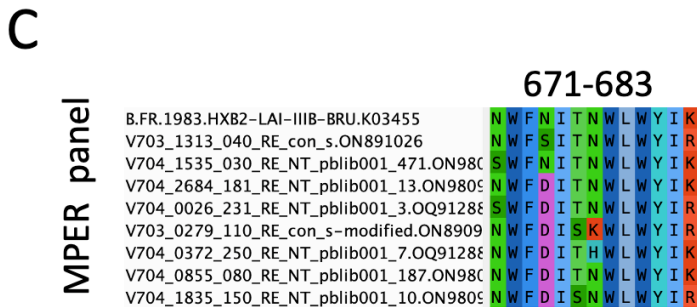

Supplement: S6 Fig — (A) The frequency of MPER sensitivity/resistance signatures as defined in Bricault et al. [34] in the class specific panel versus other AMP viruses. The height of the letter is indicative of the frequency of the amino acid in a given position in each group. An O indicates an N-linked glycosylation site. Blue are sensitivity signatures, red are resistance signatures, and black amino acids were not significantly associated with either one. (B) Shorter V1 region lengths with fewer glycans were associated with MPER sensitivity [34], and these were not enriched in the MPER sensitive panel. (C) The sequence alignment for the CD4bs bnAb sensitive panel across the epitope region. This a highly conserved epitope. (PDF) [file ppat.1013739.s006.pdf]
