# Supplementary material for: Contemporary HIV-1 envelope pseudovirus panels for detecting and assessing B cell lineages with broadly neutralizing antibody potential
Source: PLoS Pathog. 2026 Apr 23;22(4):e1013739. doi: 10.1371/journal.ppat.1013739 (PMC13105343; doi:10.1371/journal.ppat.1013739)
Supplement: S5 Fig — (A) The frequency of CD4bs bnAb sensitivity/resistance signatures as defined in Bricault et al. [34] in the class specific panel versus other AMP viruses. The height of the letter is indicative of the frequency of the amino acid in a given position in each group. An O indicates an N-linked glycosylation site. Blue are sensitivity signatures, red are resistance signatures, and black amino acids were not significantly associated with either one. (B) Shorter V1 + V2 region lengths with fewer glycans were associated with CD4bs bnAb sensitivity [34], and while shorter combined V1V2 lengths were not enriched in the sensitive panel, fewer glycans in the V1V2 loop regions were. Shorter V5 loops with fewer glycans were also previously associated with enhanced CD4bs bnAb sensitivity [34], and both of these characteristics were enriched in the sensitive panel. (C) The sequence alignment for the CD4bs bnAb sensitive panel across the epitope region. (PDF) [file ppat.1013739.s005.pdf]

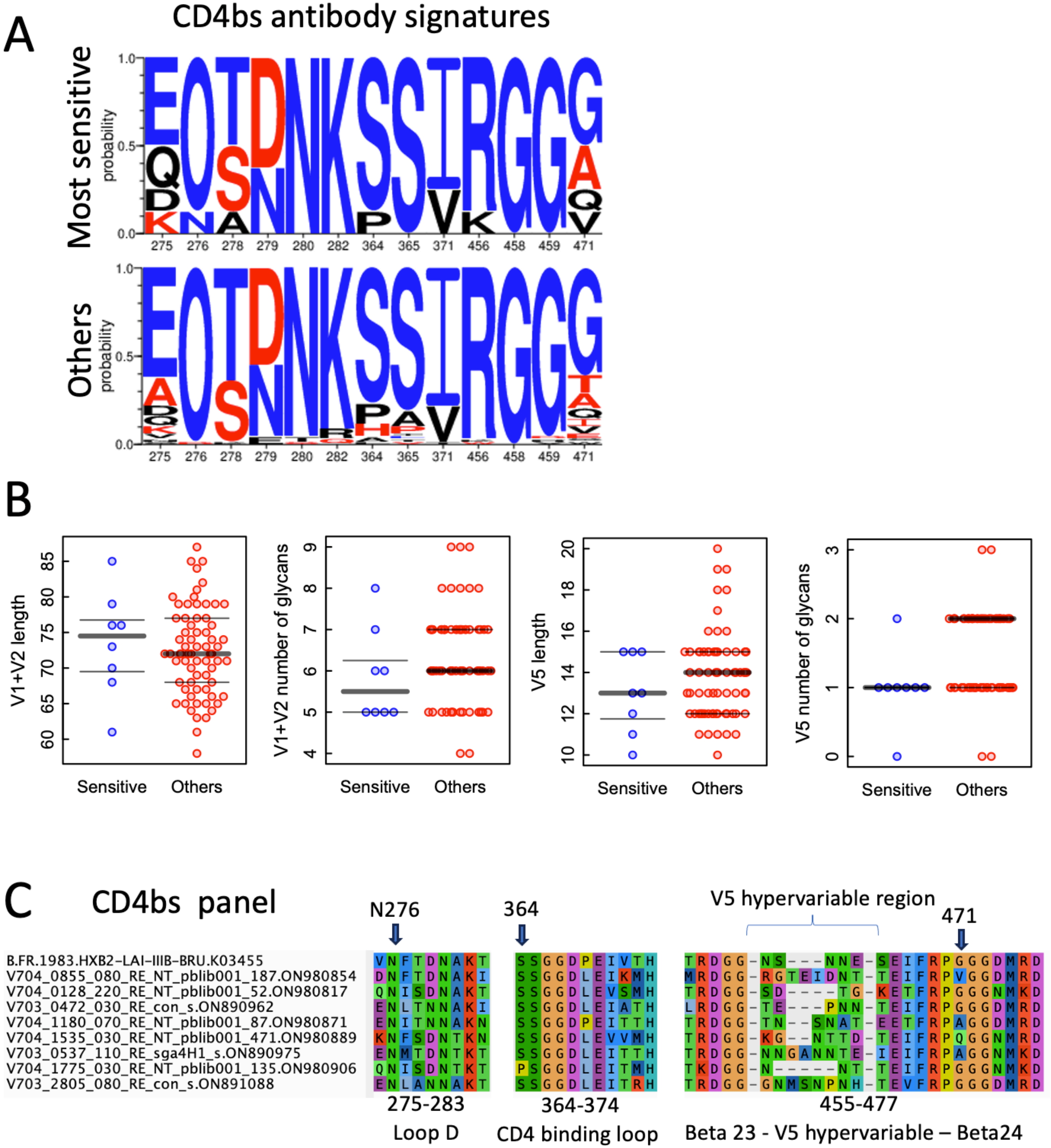
A
CD4bs antibody signatures
Most sensitive
Others
probability
1.0
0.5
0.0
275 276 278 279 280 282 364 365 371 456 458 459 471
B
V1+V2 length
V1+V2 number of glycans
V5 length
V5 number of glycans
Sensitive
Others
C
CD4bs panel
N276
364
V5 hypervariable region
471
B.FR.1983.HXB2-LAI-IIIB-BRU.K03455 VNFTDNAKT SSGGDPEIVTH TRDGG-NS---NNE-SEIFRPGGGDMRD
V704_0855_080_RE_NT_pblib001_187.ON980854 DNFTDNAKI SSGGDLEIKMH MRDGG-RGTEIDNT-TEIFRPVGGDMRD
V704_0128_220_RE_NT_pblib001_52.ON980817 QNISDNAKT SSGGDLEVSMH TRDGG-SD----TG-KETFRPGGGNMKD
V703_0472_030_RE_con_s.ON890962 ENLTNNAKI SSGGDLEIATH LRDGG-TE---PNN-TEIFRPGGGDMRD
V704_1180_070_RE_NT_pblib001_87.ON980871 ENITNNAKN SSGGDPEITTH TRDGG-TN--SNAT-EETFRPAGGDMRD
V704_1535_030_RE_NT_pblib001_471.ON980889 KNFSDNTKN SSGGDLEVVMH TRDGG-KG--NTTD-TEIFRPQGGNMRD
V703_0537_110_RE_sga4H1_s.ON890975 ENMTDNTKT SSGGDLEITTH TRDGG-NNGANNTE-IEIFRPAGGNMKD
V704_1775_030_RE_NT_pblib001_135.ON980906 QNISDNTKT PSGGDLEITMH TKDGG-N------NT-TEIFRPGGGNMKD
V703_2805_080_RE_con_s.ON891088 ENLANNAKT SSGGDLEITRH TRDGG-GNMSNPNH-TEVFRPGGGDMRD
275-283
Loop D
364-374
CD4 binding loop
455-477
Beta 23 - V5 hypervariable – Beta24
