# Supplementary material for: Contemporary HIV-1 envelope pseudovirus panels for detecting and assessing B cell lineages with broadly neutralizing antibody potential
Source: PLoS Pathog. 2026 Apr 23;22(4):e1013739. doi: 10.1371/journal.ppat.1013739 (PMC13105343; doi:10.1371/journal.ppat.1013739)
Supplement: Attachment — Submitted filename: PPATHOGENS_response.BK.v1.docx [file ppat.1013739.s016.docx]

**Reviewers' Comments:**

Reviewer's Responses to Questions

Authors Responses in Blue

**Part I - Summary**
Please use this section to discuss strengths/weaknesses of study, novelty/significance, general execution and scholarship.

Reviewer #1: Success of HIV-1 vaccinations in pre-clinical and clinical trials is assessed using stringent Tier-2 HIV-1 virus neutralizing assays that tend to represent global diversity of circulating strains. However, current panels of viruses used in standardized assays were derived from strains/envelopes harvested almost 2 decades ago.

Here, Betty Korber et al, report the evolution of HIV-1 circulating strains in regards with global diversity of Envelopes, recombinant forms and hence, reduced value in assessing impact of vaccine induced immunity. Using transmitted founder virus strains from an Antibody Mediated Protection (AMP) trials, HVTN 703 and 704, the authors show the value of updated pseudovirus panels that can highlight improved representation of contemporary virus strains/panels that need to be periodically updated to support vaccine studies or HIV-1 vaccine outcomes in general.

The manuscript is very well written, and the data is compelling that designing new panels to quantify HIV-1 neutralizing capability in vitro, such as with the SHEP-T2 and the REP-T2 can substantially support HIV-1 vaccine trials as well as understanding elicitation of early breadth using germline targeted and shepherding approaches.

I Recommend publication of this exciting advance in assessing HIV-1 vaccination trials.

We thank the reviewer for the positive comments and feedback.

I only have one minor comment in the supplementary tables.

Minor Comments.
1. The headers needed for the tables in Table S1A is only included in Table S1B (IC50, and targeted Env regions such as CD4bs, V3, V2,FP, MPER)

Thank you. We have fixed the issue.

Reviewer #2: Korber et al have conducted an insightful follow-up study building on previous research regarding the efficacy trials of Antibody Mediated Prevention (AMP). They underscore the importance of refreshing the reference panel of HIV-1 Envelope pseudotyped viruses that are routinely used to evaluate broadly neutralizing antibodies (bNab), as well as to assess the quality of antibody responses prompted by vaccines. Given the passage of time since the original panel was established and the ongoing evolution of HIV-1, this study is both timely and necessary.

Panels that incorporate current and recently transmitted HIV-1 strains have the potential to provide greater insights into bNab development and improve the design of therapeutic regimens. By utilizing envelopes from participants who received placebos in the AMP efficacy trials, the authors have developed panels that can detect early neutralizing responses and class-specific broadly neutralizing antibodies, along with antibody responses to contemporary and naturally circulating variants. While these panels may not be perfect (lacking representation from all global clades), they offer valuable tools for investigating bNab in various contexts.

Thus, this study is significant and will help advance the field of broadly neutralizing antibodies for the prevention and treatment of HIV-1. The methodology applied mirrors that of the previously established reference panel, originally developed from strains collected between 1998 and 2010, which served as a comparator for the new panels created. Therefore, there are no major issues with this study. However, below are some minor points to address for clarity and consistency.

We thank the reviewer for the positive comments and feedback.

**Part II – Major Issues: Key Experiments Required for Acceptance**
Please use this section to detail the key new experiments or modifications of existing experiments that should be absolutely required to validate study conclusions.

Generally, there should be no more than 3 such required experiments or major modifications for a "Major Revision" recommendation. If more than 3 experiments are necessary to validate the study conclusions, then you are encouraged to recommend "Reject".

Reviewer #1: None except spell checks through the manuscript.

Reviewer #2: None

**Part III – Minor Issues: Editorial and Data Presentation Modifications**
Please use this section for editorial suggestions as well as relatively minor modifications of existing data that would enhance clarity.

Reviewer #1: Minor Comments.
1. The headers needed for the tables in Table S1A is only included in Table S1B (IC50, and targeted Env regions such as CD4bs, V3, V2,FP, MPER)

Thank you. We have fixed the issue for both tables S1A and S1C.

Reviewer #2: Another thorough read
1.- Extra words
- Line 388 in Discussion, “and for even for detecting,” one of the “for” should be deleted.
- Line 562 in Figure legend 1, “longer branch lengths within both and B and C clades,” one of the “and” should be deleted.

Thank you for catching these typos, we have now corrected both.

2.- Mostly in the Discussion and Materials and Methods, there is inconsistent formatting for figures and tables. Sometimes bold, sometimes not.
- Lines 386, 387, 395, 411, 417, 480, 481, 500, and 510.

We apologize for the inconsistency. We have now used the journals recommended format for citing figures throughout the text.

Figure legends and Supplemental tables
1.- Main figure legends have a lot of discussion of the results instead of description of the figure/analysis, thus there are similar sentences in the Results section and Figure legends, making the paper very repetitive.

Thank you for the thought, but we believe that by including a brief summary in the figure captions it helps frame the context for the reader, and we prefer to leave them as written if it is OK with the editor.

2.- The Supplemental Tables are not described chronologically. The authors start with Table S2, then go to Table S5, then Tables S1, S3 and S4. In addition, Table S8 is mentioned several times and there is no Table S8 in the manuscript.

Thank you for catching this! References to Tables S1 and S2 were in the correct order, but we did remove an out-of-order reference to Table S5 and fixed the ordering problem the reviewer noticed.

Regarding Table S8: We apologize for this oversite and appreciate that the reviewer caught it! Table S8 was only included in the data repository. We have now included it in the supplementary files.

**Figure resubmission:**

While revising your submission, we strongly recommend that you use PLOS’s NAAS tool ([https://ngplosjournals.pagemajik.ai/artanalysis](https://urldefense.com/v3/__https:/ngplosjournals.pagemajik.ai/artanalysis__;!!GuAItXPztq0!glpZPFvQ82ffJi7dPBDAOK7-Hjk94zoZMbrYmBZlw5K-2zcgE7d2OAFUyxrlQF0zWhkyfJtiQzZ9pdF_9A$)) to test your figure files. NAAS can convert your figure files to the TIFF file type and meet basic requirements (such as print size, resolution), or provide you with a report on issues that do not meet our requirements and that NAAS cannot fix.

After uploading your figures to PLOS’s NAAS tool - [https://ngplosjournals.pagemajik.ai/artanalysis](https://urldefense.com/v3/__https:/ngplosjournals.pagemajik.ai/artanalysis__;!!GuAItXPztq0!glpZPFvQ82ffJi7dPBDAOK7-Hjk94zoZMbrYmBZlw5K-2zcgE7d2OAFUyxrlQF0zWhkyfJtiQzZ9pdF_9A$), NAAS will process the files provided and display the results in the "Uploaded Files" section of the page as the processing is complete. If the uploaded figures meet our requirements (or NAAS is able to fix the files to meet our requirements), the figure will be marked as "fixed" above. If NAAS is unable to fix the files, a red "failed" label will appear above. When NAAS has confirmed that the figure files meet our requirements, please download the file via the download option, and include these NAAS processed figure files when submitting your revised manuscript.

All final form of each figure was generated using the PLOS’s NAAS tool and provided as NAAS tif output.

**Reproducibility:**

To enhance the reproducibility of your results, we recommend that authors of applicable studies deposit laboratory protocols in protocols.io, where a protocol can be assigned its own identifier (DOI) such that it can be cited independently in the future. Additionally, PLOS ONE offers an option to publish peer-reviewed clinical study protocols. Read more information on sharing protocols at [https://plos.org/protocols?utm_medium=editorial-email&utm_source=authorletters&utm_campaign=protocols](https://urldefense.com/v3/__https:/plos.org/protocols?utm_medium=editorial-email&utm_source=authorletters&utm_campaign=protocols__;!!GuAItXPztq0!glpZPFvQ82ffJi7dPBDAOK7-Hjk94zoZMbrYmBZlw5K-2zcgE7d2OAFUyxrlQF0zWhkyfJtiQzZn7Ni2nQ$)

Currently the detailed protocols are maintained in the HIV-1 Immunology database, as they have been for many years. This documentation is extensive. At this point we prefer to leave them there, as most of the people in our field who used these protocols know to look for them there, as well as to look for any updates and advances there. We already had included the link in the methods:

<http://www.hiv.lanl.gov/content/nab-reference-strains/html/home.htm>
